# Integrated analysis of the DNA methylome and RNA transcriptome during the development of skeletal muscle in Duroc pigs

**DOI:** 10.1186/s12864-024-10404-0

**Published:** 2024-05-22

**Authors:** Shi-yin Li, Yun-zhou Wang, Wei Chen, Li-xia Ma, Jian-min Zhang, Yu-lun Zhang, Yong-qing Zeng

**Affiliations:** 1 Shandong Provincial Key Laboratory of Animal Biotechnology and Disease Control and Prevention, College of Animal Science and Technology, Shandong Agricultural University, Street, Taian, 271018 Shandong China; 2 Shandong Vocational Animal Science and Veterinary College, Weifang, 261061 Shandong China; 3 Department, Shandong Ding Tai Animal Husbandry Co. Ltd., Jinan, 250300 Shandong China

**Keywords:** Duroc pigs, WGBS, DNA methylome, Transcriptome, Skeletal muscle

## Abstract

**Background:**

Skeletal muscle development plays a crucial role in yield and quality of pork; however, this process is influenced by various factors. In this study, we employed whole-genome bisulfite sequencing (WGBS) and transcriptome sequencing to comprehensively investigate the longissimus dorsi muscle (LDM), aiming to identify key genes that impact the growth and development of Duroc pigs with different average daily gains (ADGs).

**Results:**

Eight pigs were selected and divided into two groups based on ADGs: H (774.89 g) group and L (658.77 g) group. Each pair of the H and L groups were half-siblings. The results of methylation sequencing revealed 2631 differentially methylated genes (DMGs) involved in metabolic processes, signalling, insulin secretion, and other biological activities. Furthermore, a joint analysis was conducted on these DMGs and the differentially expressed genes (DEGs) obtained from transcriptome sequencing of the same individual. This analysis identified 316 differentially methylated and differentially expressed genes (DMEGs), including 18 DMEGs in promoter regions and 294 DMEGs in gene body regions. Finally, LPAR1 and MEF2C were selected as candidate genes associated with muscle development. Bisulfite sequencing PCR (BSP) and quantitative real-time PCR (qRT–PCR) revealed that the promoter region of LPAR1 exhibited significantly lower methylation levels (*P* < 0.05) and greater expression levels (*P* < 0.05) in the H group than in the L group. Additionally, hypermethylation was observed in the gene body region of MEF2C, as was a low expression level, in the H group (*P* < 0.05).

**Conclusions:**

These results suggest that the differences in the ADGs of Duroc pigs fed the same diet may be influenced by the methylation levels and expression levels of genes related to skeletal muscle development.

**Supplementary Information:**

The online version contains supplementary material available at 10.1186/s12864-024-10404-0.

## Introduction

The Duroc pig, which was first discovered in South America in the 1960s, has rapidly become popular and blue is one of the top lean pig breeds worldwide [1]. This species has outstanding qualities, such as a rapid growth rate, high feed conversion efficiency, and excellent meat yield [2–4], and is often used as a terminal sire when fattening pigs are produced. However, the growth performance of Duroc pigs gradually declines after they reach 110 kg body weight. Selecting Duroc pigs that can maintain good growth and meat production performance from 110 kg to 130 kg will substantially increase the economic benefits of pig farming.

The largest tissue in mammals, skeletal muscle, which comprises 40% of the body, is essential for locomotor activity, energy expenditure, and meat production performance [5, 6]. The amount and quality of meat produced are strongly based on the development and physical properties of the muscle since it is a crucial end product of livestock production [7]. Therefore, exploring the mechanisms underlying skeletal muscle growth and development is essential for increasing livestock production performance.

However, skeletal muscle development is influenced by genetic and nutritional factors and a range of intricate epigenetic regulatory mechanisms, such as DNA methylation. DNA methylation is an epigenetic marker that is crucial for regulating genomic function and maintaining normal mammalian development by controlling gene activity and other regulatory factors [8, 9]. As research on DNA methylation has intensified, numerous studies have highlighted its impact on muscle growth and development in livestock [10–12]. DNA methylation levels vary throughout different stages of skeletal muscle development in pigs and gradually decrease as pig embryos grow and develop [13]. Furthermore, DNA methylation differs across pig breeds, revealing correlations with skeletal muscle growth and development [14, 15]. However, further DNA methylation studies in siblings or half-siblings of pigs are needed.

This study aimed to achieve the following objectives: 1) To select the LDM of sibling and half-sibling Duroc pigs and perform a comprehensive analysis of the global DNA methylation levels in the H (high ADG) and L (low ADG) groups using WGBS (whole-genome bisulfite sequencing). 2) To identify DMRs (differentially methylated regions) and DMGs (DMR-associated genes) in the two comparison groups. 3) To analyse the DNA methylome and transcriptome of LDMs in Duroc pigs, identify genes associated with growth and development, and determine their expression and methylation levels.

## Results

### Genome-wide DNA methylation profiling and patterns

To investigate the DNA methylation patterns of LDMs in Duroc pigs, we utilized single-base resolution WGBS [16] to assess global DNA methylation levels in both the H and L groups. After removing adapter contaminants, low-quality reads, and reads containing Ns, we collected 490,435,608 to 669,644,232 clean reads from each of the eight DNA library samples (Table 1). The mapping ratio of clean reads to the Duroc pig genome ranged from 86.45% to 87.26% for all eight models. The sequencing data were ready for further analysis, with sequencing depths reaching 28.01 (H) and 31.36 (L). The bisulfite conversion efficiency ranged from 99.00% to 99.04% per sample (Table 1).

**Table 1:** Summary of genome-wide methylation sequencing data

Samples	Clean reads	Mapped reads	Mapped ratio (%)	Genome size	Sequence depth	BS Conversion (%)
H1	595191668	519358211	87.26	2488630688	31.3	99.04%
H2	490435608	427408324	87.15	2488630688	25.76	99.02%
H3	514939188	446151742	86.64	2488630688	26.89	99.02%
H4	536252010	466334203	86.96	2488630688	28.11	99.00%
L1	525286770	454122700	86.45	2488630688	27.37	99.02%
L2	669644232	580918741	86.75	2488630688	35.01	99.03%
L3	600987034	522532244	86.95	2488630688	31.5	99.02%
L4	605375476	523792647	86.52	2488630688	31.57	99.04%

**Fig. 1:**
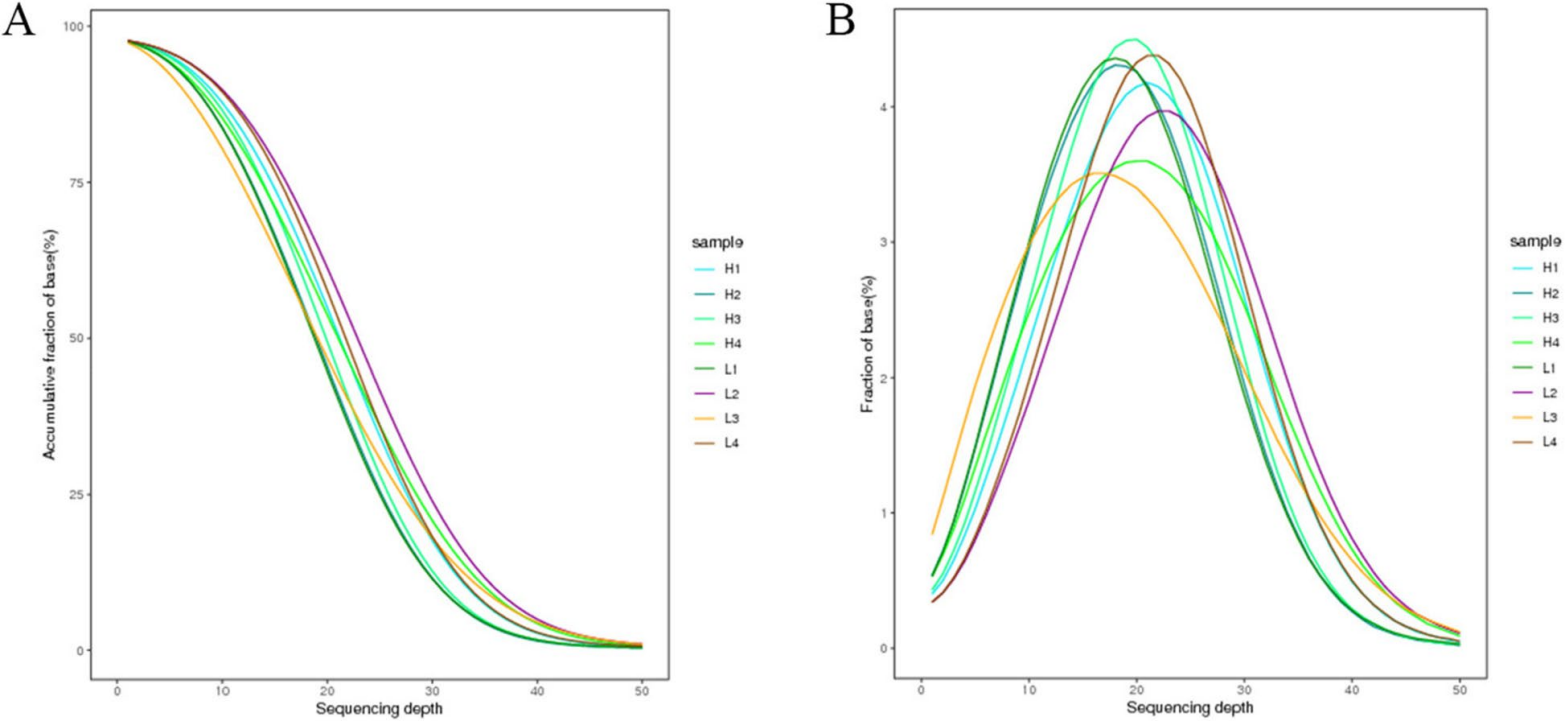
**A** Cumulative genomic distribution of the H (high ADG) and L (low ADG) groups of LDMs of Duroc pigs at 110-130 kg. **B** Genome coverage distribution of the H and L groups

Over 80% of the whole-genome loci had a coverage depth of 10x or more (Fig. 1A), with most of the loci having a coverage depth of 10-30x, resulting in a standard distribution (Fig. 1B). These findings indicated high coverage of individual loci, a wide sequencing range, and credible results.

**Fig. 2:**
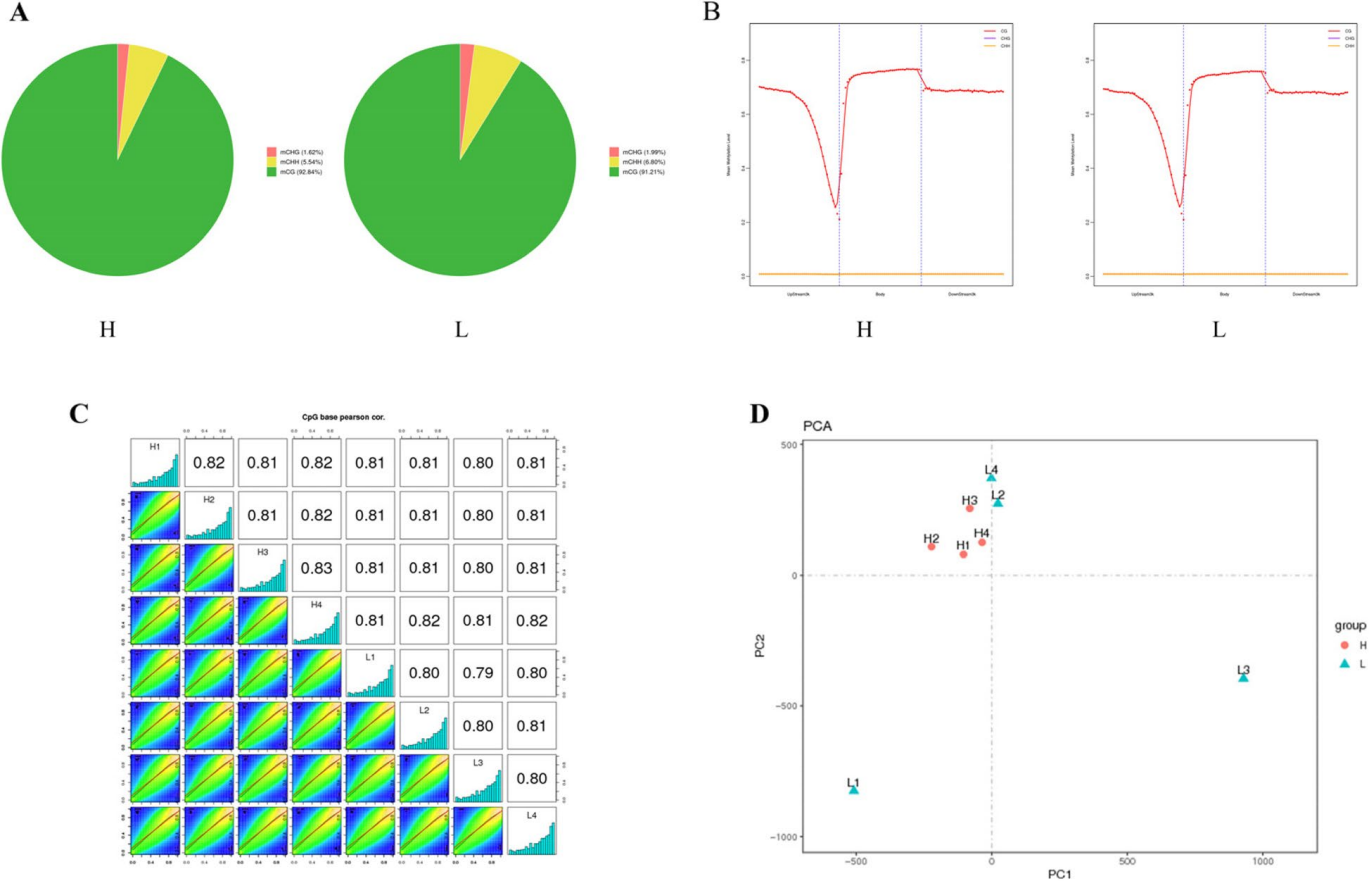
DNA methylation patterns in Duroc pig LDMs. **A** The proportions of mCs (mCG, mCHG, and mCHH) in the H and L groups. **B** The mCG, mCHG, and mCHH methylation levels in different sequence regions in the H and L groups. **C** Correlation analysis of methylation levels between samples from the two groups. **D** Principal component analysis of all samples

Our investigation of the distribution of mCs in three sequence contexts revealed that CG methylation was the most prevalent form of methylation in all the samples, occurring less frequently in the CHH and CHG sequences (Fig. 2A). The proportions of these three methylation forms remained relatively stable across all eight samples. While CG methylation levels were greater than 90% in most examples, CHG and CHH methylation levels ranged between 1.62% and 6.80% (Fig. 2A). Furthermore, to examine the methylation distribution in different genetic regions, we evaluated the methylation levels of mCG, mCHG, and mCHH in the gene-body, upstream, and downstream areas of the H and L groups. On a genome-wide scale, we observed similar methylation profiles for mCHG/CHG and mCHH/CHH in all regions (Fig. 2B). However, from the upstream region to the gene body and downstream areas, the methylation level of mCG decreased and then increased before levelling off.

Pearson correlation analysis and Principal Component Analysis (PCA) were used to assess the similarity of the eight LDM samples. Pearson correlation analysis of the CpG bases suggested that a high correlation across all samples(r> 0.79) (Fig. 2C). Furthermore, PCA revealed no significant distinction between the sample groups, as they did not form separate clusters (Fig. 2D).

### Identification of DMRs

**Fig. 3:**
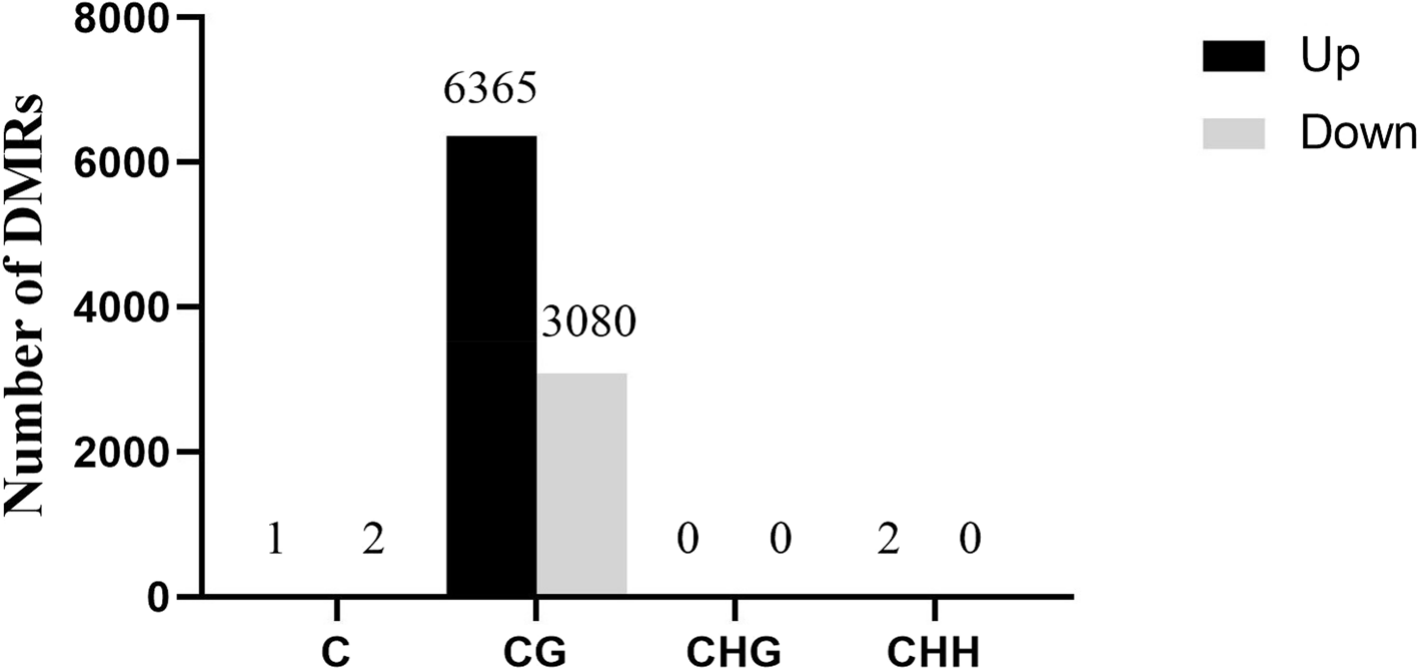
Number of DMRs in the H vs L group (*P* < 0.05)

The genomic regions with different DNA methylation levels in the H and L groups were identified. In total, we identified 9450 DMRs: 6368 hyper-DMRs and 3082 hypo-DMRs (Fig. 3). Among the mCG methylation types, 6365 DMRs were increased, and 3080 DMRs were decreased. For the mCHG methylation type, only one DMR was decreased. Among the mCHH methylation types, two DMRs were increased, and eight were decreased. Finally, for the mC methylation types, one DMR was increased, and two DMRs were decreased. Detailed information on these DMRs is shown in Supplementary Table 1.

**Table 2:** Primers used in the study

Gene	Primer sequence	Purpose	Product size(bp)
Lpar1-BSP-F	5’-GAATATTTTTTGAGAAGTTTGAGAAGTTT-3’	Identification of	209
Lpar1-BSP-R	5’-CCATAACAACAATATAACAAAAAAATTAAC-3’	methylation level	
Mef2c-BSP-F	5’-TATGTGTGTTTTATAGTATTATTTTTTGTTTT-3’	Identification of	358
Mef2c-BSP-R	5’-ATCTCCTAATAAACTTAAATTTTACAAAATTA-3’	methylation leveL	
Lpar1-F	5’-GGAAAGTACCTTGCCACAGAA-3’	qRT-PCR	129
Lpar1-R	5’-GAAGCGGCGGTTGACATA-3’		
Mef2c-F	5’-GAGCGTGCTGTGTGACTGTGAG-3’	qRT-PCR	82
Mef2c-R	5’-CATGTCCGTGCTGGCATACTGG-3’		
Gapdh-F	5’-AAAGGCCATCACCATCTTCC-3’	qRT-PCR	135
Gapdh-R	5’-GCCCCACCCTTCAAGTGAGCC-3’		

**Fig. 4:**
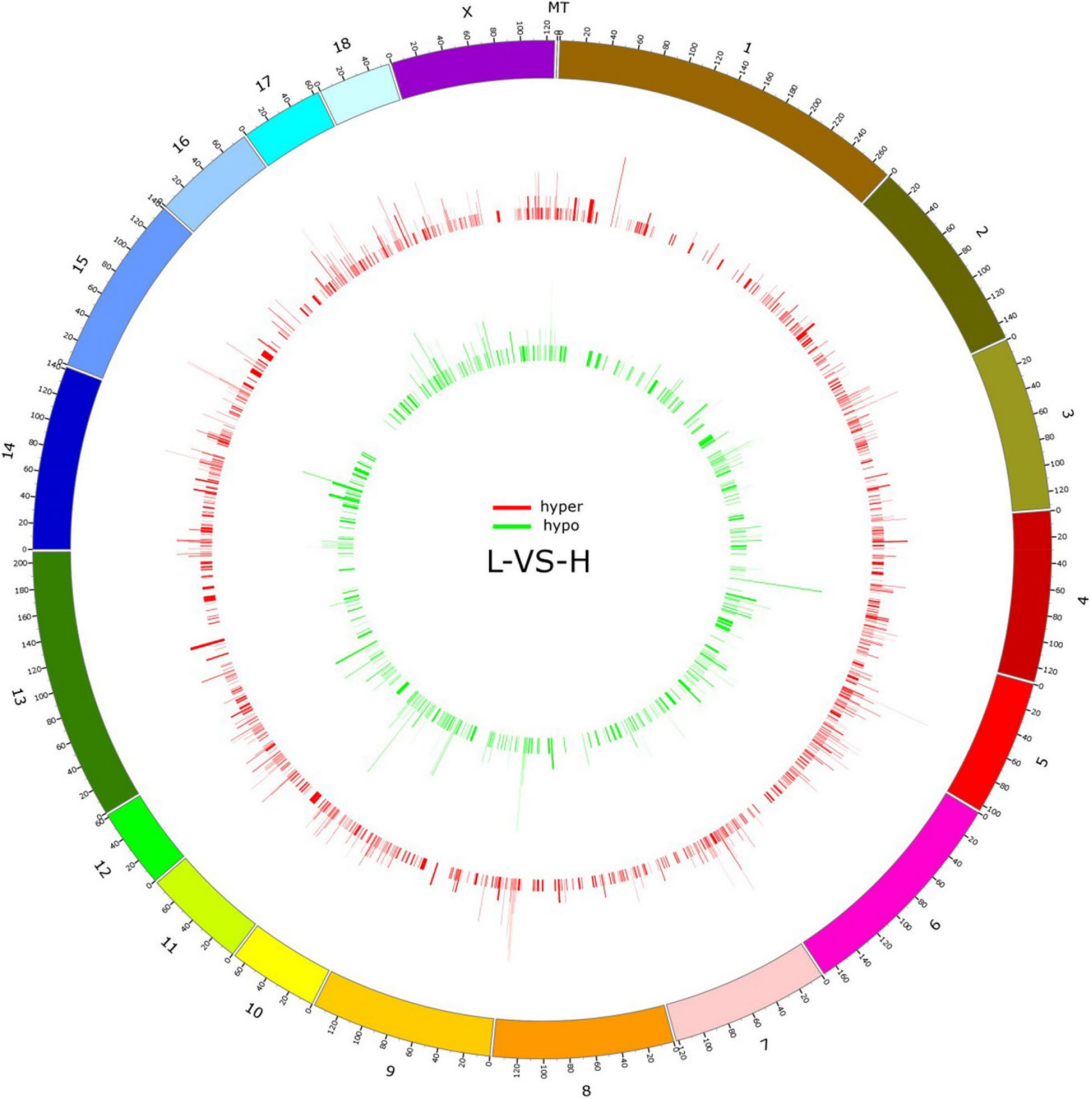
Distribution of DNA methylation on the Duroc pig chromosome. DMR density was calculated in 100-kb windows across the genome. The height of the column indicates the number of DMRs in each window. Hyper-DMRs are shown in the red column. Hypo-DMRs are shown as green columns

To investigate the distribution of differential methylation across the genome, we calculated the density of DMRs in 100-kb windows (Fig. 4). Our analysis revealed the presence of DMRs on each chromosome. Specifically, among the 9445 DMRs (mCG methylation type) (*P* < 0.05, difference $\ge 15\%$) in the H vs L group, 6365 hyper-DMRs showed the greatest enrichment on chromosomes 6 (NC_010448.4), 1 (NC_010443.5), and X (NC_010461.5). In contrast, 3080 hypo-DMRs showed the greatest enrichment on chromosomes 1 (NC_010443.5), 6 (NC_010448.4), and X (NC_010461.5).

### GO and KEGG pathway enrichment analysis of DMR-related genes

**Fig. 5:**
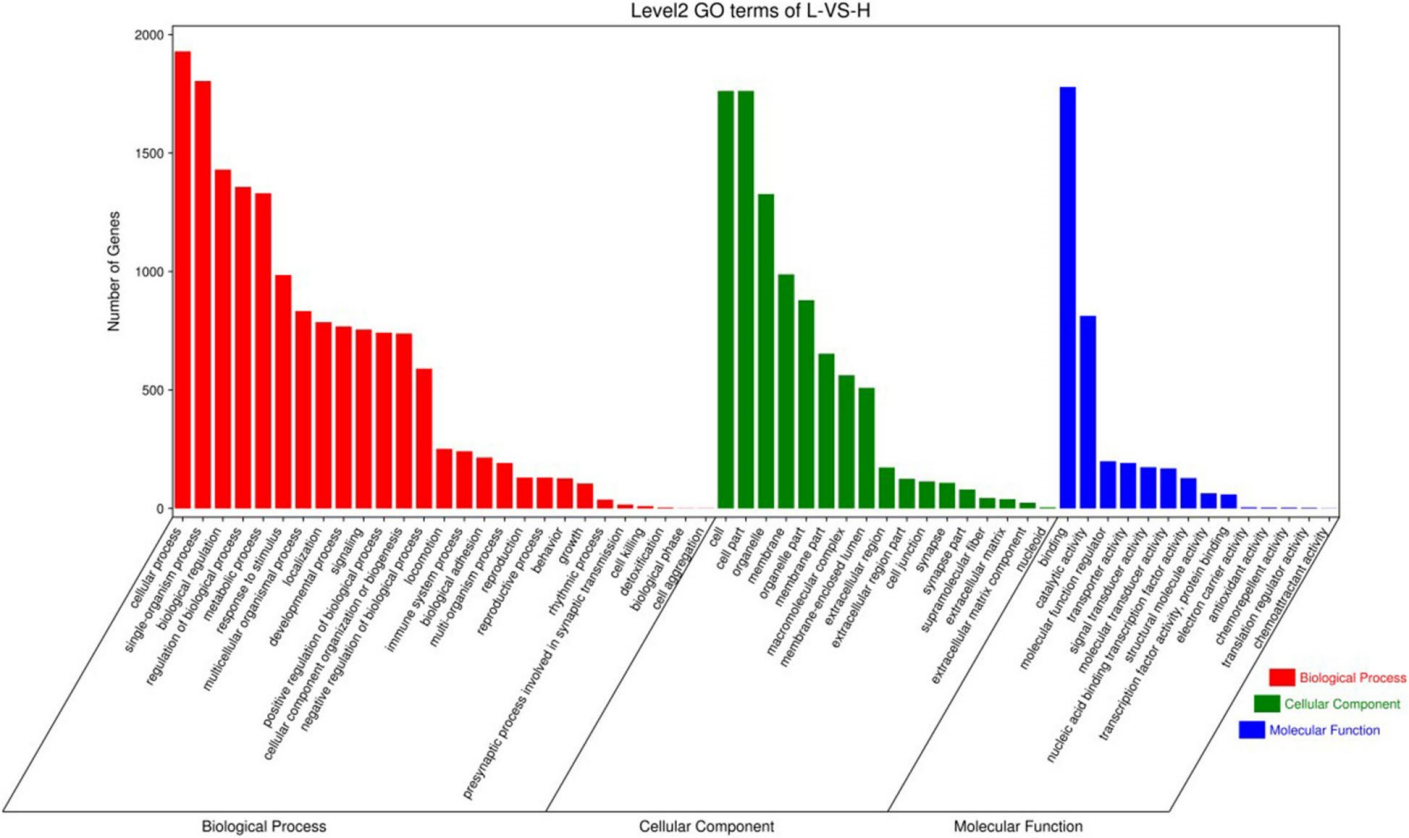
GO enrichment analysis of DMGs (those were annotated to different GO terms in biological process, cellular component, and molecular function) in the H vs L group

**Fig. 6:**
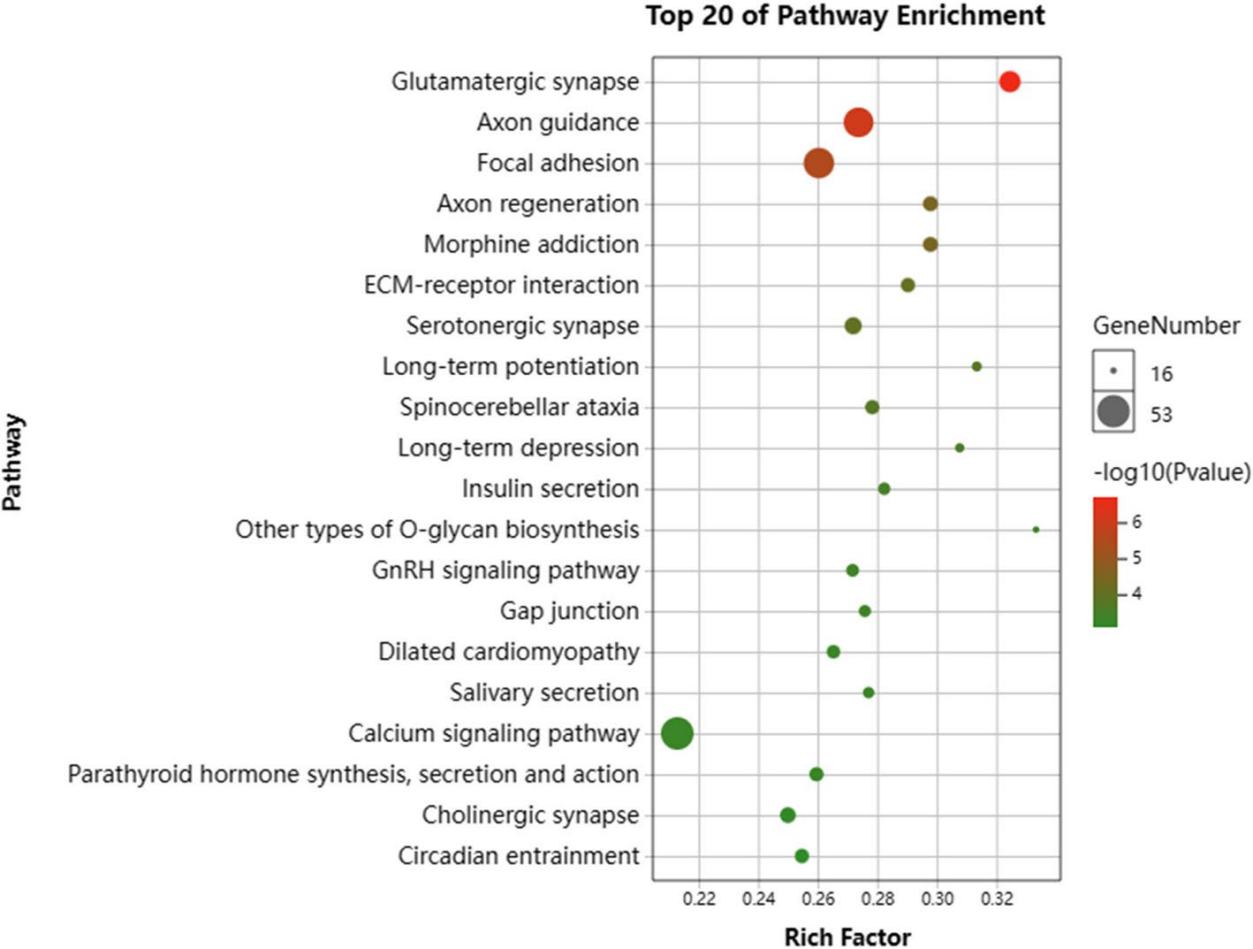
KEGG enrichment analysis of DMGs (those were annotated to different pathways) in the H vs L group

We performed GO and KEGG pathway enrichment analyses to determine the potential functions of the DMGs. Our results showed that the DMGs were significantly enriched in several GO terms related to biological processes, cellular components, and molecular functions (Fig. 5 and Supplementary Table 2). Specifically, the top ten enriched GO terms were cell (1762), cell part (1762), anatomical structure development (726), single-organism developmental process (763), developmental process (768), plasma membrane (567), cell periphery (581), binding (1779), cellular process (1929), single-multicellular organism process (762) and system development (593). In addition, we found that the DMGs were significantly enriched in several GO terms associated with muscle development, including actin cytoskeleton organization (112), actin filament-based process (120), muscle structure development (82), actin filament organization (69), and muscle tissue development (60). Furthermore, KEGG pathway enrichment analysis was performed. A total of 45 KEGG terms were significantly enriched (Fig. 6, Supplementary Table 3). The five most highly represented pathways were glutamatergic synapses (37), axon guidance (49), focal adhesion (50), axon regeneration (28), and morphine addiction (28).

### Integrated analysis of DMGs and DEGs

**Fig. 7:**
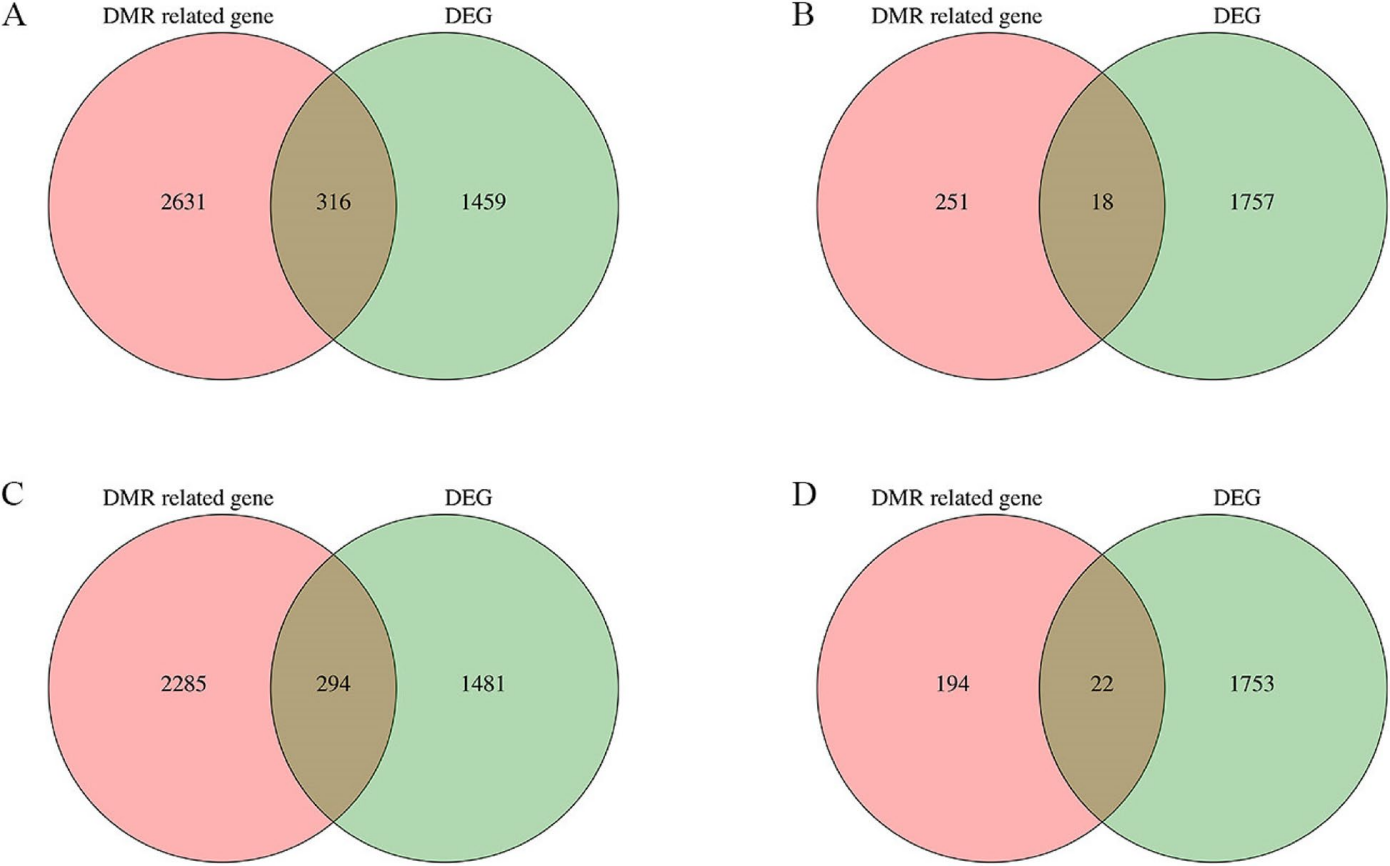
Venn diagram analysis of DMGs and DEGs. **A** The number of overlapping DEGs in the DNA methylome and transcriptome. **B** The number of overlapping DEGs upstream. **C** The number of overlapping DEGs in the gene body. **D** The number of overlapping DEGs downstream. All DEGs were determined based on statistical significance at < 0.05

To further elucidate the relationship between DNA methylation and gene expression during late growth and development in Duroc pigs, we conducted a comprehensive analysis of the DNA methylome and transcriptome of the LDM in our laboratory [17]. A Venn diagram analysis of the DEGs and DMGs in the H vs L comparison group revealed 316 overlapping genes (Fig. 7A, Supplementary Table 4). Among the 316 overlapping genes, the DMRs of 18, 294, and 22 genes were located upstream (Fig. 7B), in the gene body (Fig. 7C) and downstream (Fig. 7D), respectively. For example, LPAR1 in the H group showed hypomethylation in upstream and gene-body regions compared with that in the L group. MEF2C in demonstrated greater expression and hypermethylation in the gene-body region in the H group than in the L group. Additionally, SLIT3 expression was significantly downregulated, and the methylation levels of DMRs located in the downstream region were significantly increased.

### Validation of DNA methylomic and transcriptomic data

**Fig. 8:**
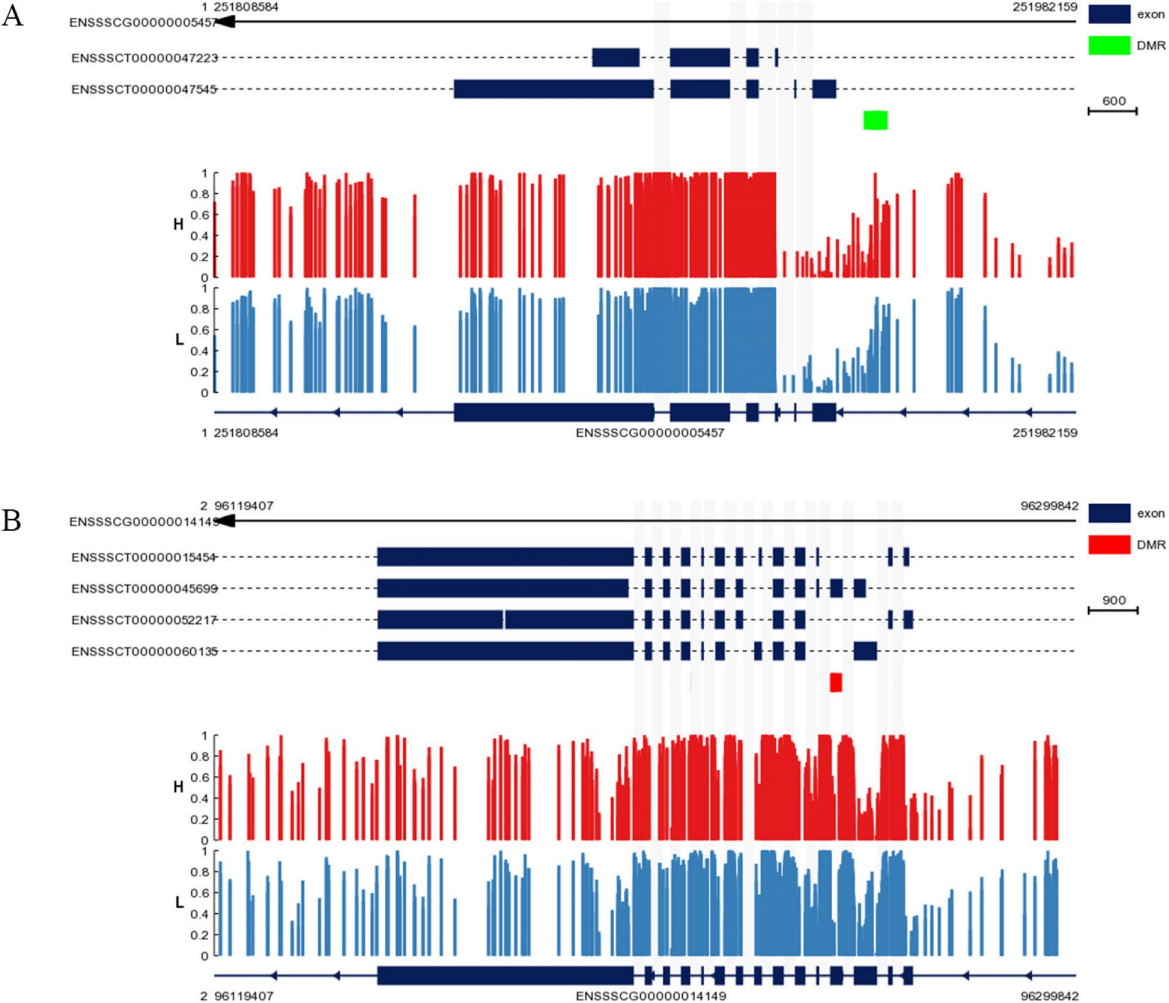
The methylation levels of LPAR1 and MEF2C in the gene region with 3 kb flanking regions of the two groups. The heights of the bars represent the methylation percentages for the H (red) and L (blue) groups. The green and red boxes indicate significant hypomethylation and hypermethylation, respectively (*P* < 0.05). The X-axes indicate the position on the scaffolds (middle). The gene structures are shown on the bottom, with the closed boxes representing exons

**Fig. 9:**
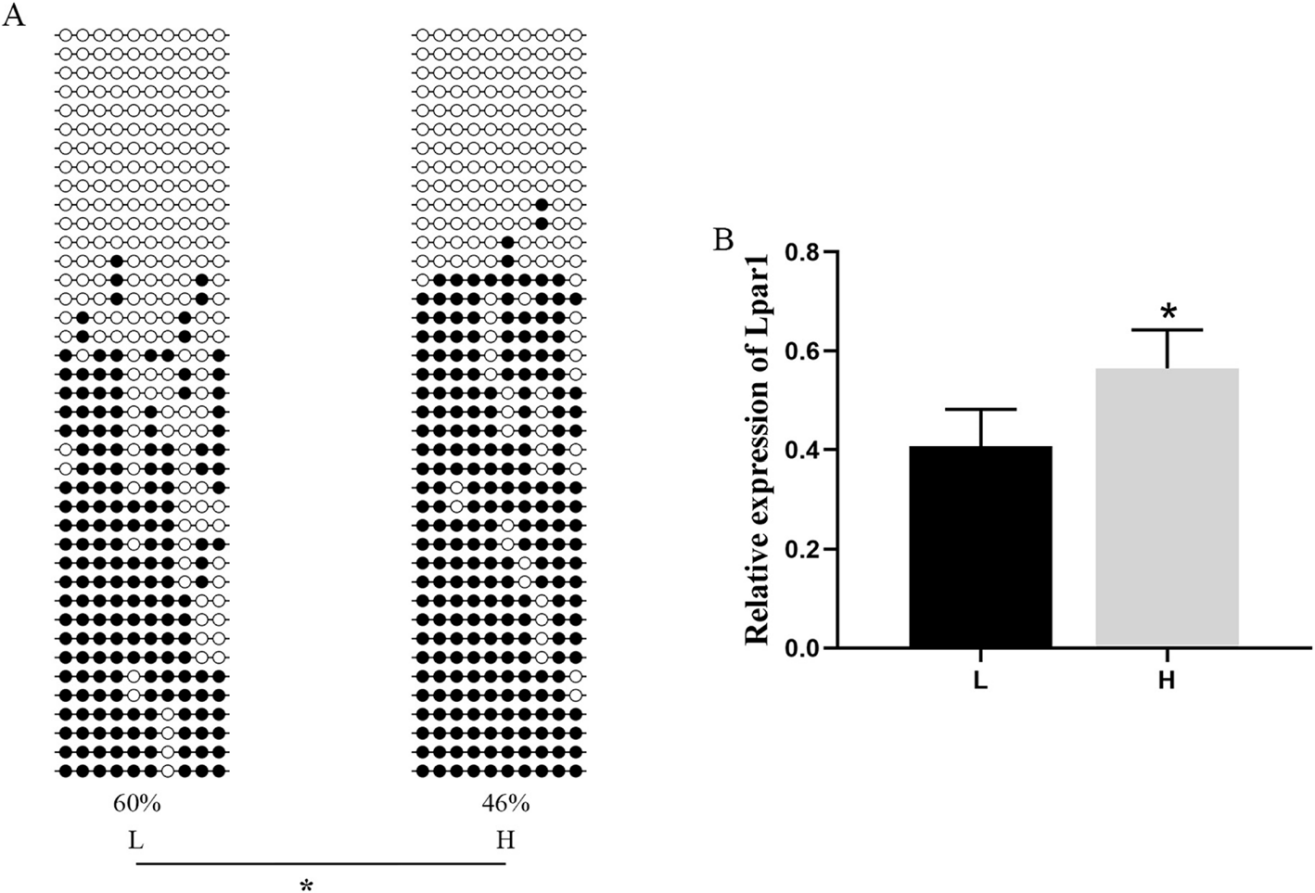
**A** Upstream methylation pattern of LPAR1 in the LDMs of Duroc pigs. Open and filled circles denote unmethylated or methylated positions, respectively. **B** The relative expression level of LPAR1 in the LDMs of Duroc pigs determined via qRT–PCR. The data are shown as the mean ± S.E. (n = 4). *, P < 0.05

**Fig. 10:**
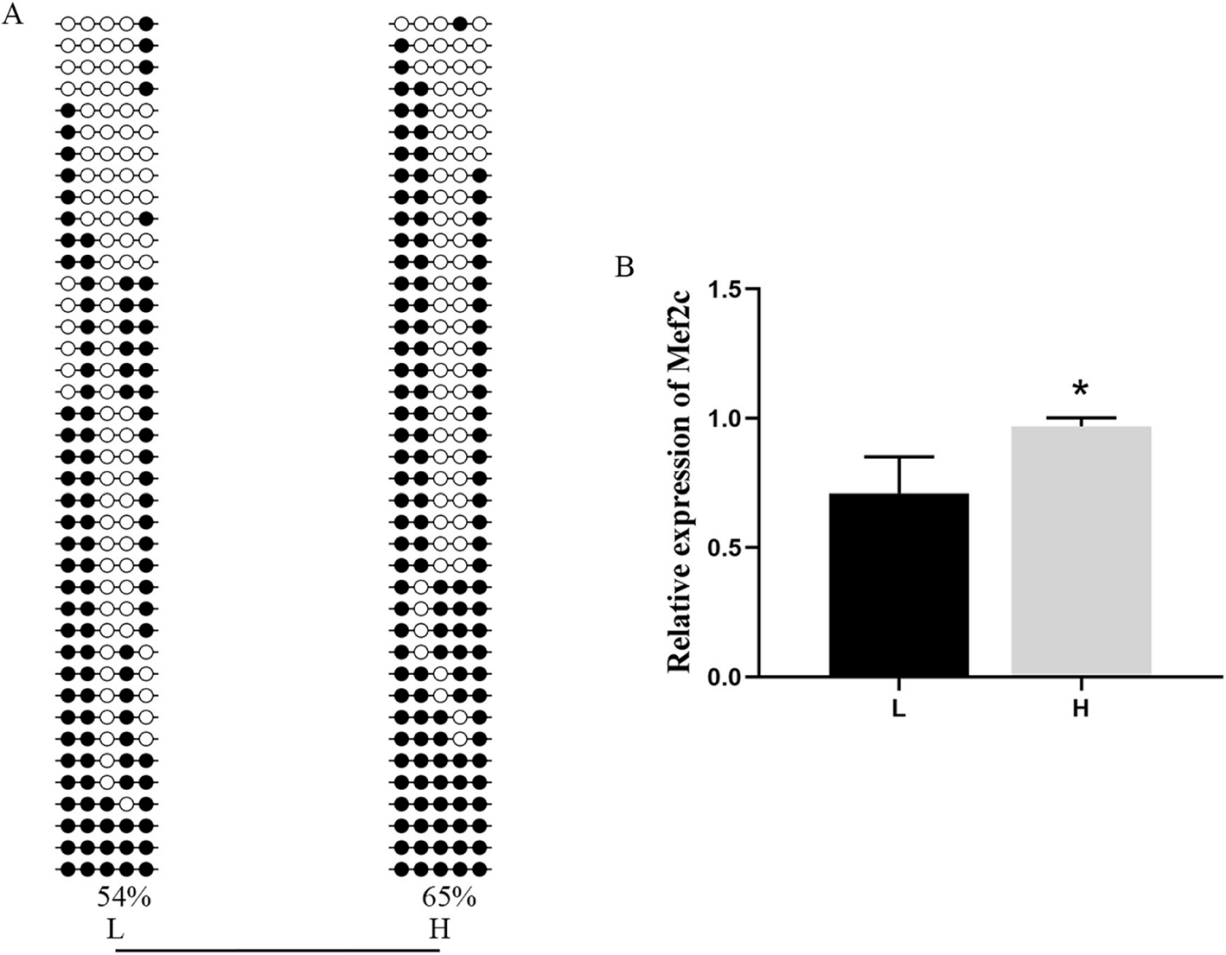
**A** Gene-body methylation pattern of MEF2C in the LDMs of Duroc pigs. Open and filled circles denote unmethylated or methylated positions, respectively. **B** The relative expression level of MEF2C in the LDMs of Duroc pigs determined by qRT–PCR. The data are shown as the mean ± S.E. (n = 4). *, *P* < 0.05

We conducted BSP (bisulfite sequencing PCR) and qRT–PCR (quantitative real-time PCR) analyses of LPAR1 and MEF2C to validate the findings from the DNA methylome and transcriptome analyses. The BSP and DNA methylome analyses confirmed that the DNA methylation level of LPAR1 in the gene promoter was significantly lower in the H group than in the L group (Figs. 8A and 9A). Additionally, qRT–PCR analysis indicated that the expression level of LPAR1 was significantly greater in the H group than in the L group (*P* < 0.05) (Fig. 9B). DNA methylome analysis revealed that the DNA methylation level of MEF2C in the gene-body region was significantly greater in the H group than in the L group (Fig. 8B). Additionally, the BSP results indicated that the DNA methylation level of MEF2C in the gene-body region was slightly greater in the H group than in the L group (Fig. 10A). Moreover, qRT–PCR analysis demonstrated that the expression level of MEF2C was significantly greater in the H group than in the L group (*P* < 0.05)(Fig. 10B).

## Discussion

ADG is considered a growth trait of pigs and an essential indicator in the process of pig production that directly affects the economic efficiency of farmers. Our laboratory previously reported the identification and functional prediction of circular RNAs, long noncoding RNAs, and mRNAs related to growth traits and skeletal muscle development in Duroc pigs with different ADGs [18]. In this study, we applied WGBS technology for methylation analysis of Duroc pigs at various growth rates to identify significant differential DMR-related genes.

The WGBS method was first proposed in 1992 [19]. This method is now widely used in DNA methylation studies in pigs because it is a high throughput, high specificity, high sensitivity, high resolution, and high coverage technique. Corbett RJ et al. used WGBS, RNA-seq, and smRNA-seq methods to identify DMRs at the stage of porcine foetal myogenesis and to validate their relationship with differentially expressed mRNAs and miRNAs [20]. In addition, a study mapped the DNA methylome in the developing pig testis via WGBS [21].

To investigate the influence of DMRs on the growth rate of Duroc pigs, we conducted methylation profiling of the LDMs and identified 9,445 DMRs. Some of these DMRs were located within genes associated with muscular development, such as MEF2C, DMD, and TGFB2 [22–24]. These findings are consistent with previous studies that have investigated DNA methylation patterns in different pig breeds and at various developmental stages. For example, Li XJ et al. compared DNA methylation profiles between Wannanhua and Yorkshire pigs and identified 58283 DMRs, including 1425 DMGs that may play a role in muscle development [15]. Similarly, Wang et al. discovered 722 DMRs and 466 DMGs, including ADCY1, AGBL4, EXOC2, FUBP3, PAPPA2, PIK3R1, MGMT, and MYH8, which are associated with muscle growth in muscle tissues of Chenghua pigs and Yorkshire pigs, from a total of 2,416,211 CpG sites [13]. Furthermore, Yang et al. generated a single-base resolution DNA methylome map of porcine skeletal muscle across 27 developmental stages using WGBS and identified more than 40,000 developmentally differentially methylated CpGs associated with muscle development genes [25]. The results from our study and those of previous studies all suggest that DNA methylation plays an important role in muscle growth and development in pigs.

Furthermore, we identified pathways associated with muscle growth and development, providing a valuable theoretical basis for further research. For example, 53 DMGs were significantly enriched in the calcium signalling pathway, 53 DMGs were increased dramatically in the MAPK signalling pathway, and 62 DMGs were substantially enriched in the PI3K-AKT signalling pathway. Interestingly, the MAPK and PI3K-AKT signalling pathways were also identified in a recent study of global DNA methylation in porcine skeletal muscle [26]. The PI3K-AKT and MAPK signalling pathways play important roles in muscle development, influencing the proliferation and differentiation of muscle cells [27, 28]. Calcium is an essential intracellular transduction signal involved in various biological functions, including myogenesis [29], muscle contraction [30], and muscular dystrophy development [31]. Overall, DNA methylation has the potential to influence the growth rate and muscle production of pigs through the pathways mentioned above.

In the present study, the BSP and qRT–PCR results showed that the H group had significantly decreased DNA methylation levels of LPAR1 in the promoter region and upregulated expression of this gene. Messmer T et al. reported that LPAR1, IGF1R, and TFRC receptor expression was upregulated in the early stages of differentiation and that supplementation with the appropriate ligands effectively induced differentiation [32]. Stimulation of the sphingosine kinase and sphingosine 1-phosphate pathways by LPAR1 significantly increased the migration of skeletal muscle cells [33]. A study conducted by Ray R et al. revealed that suppressing the LPAR1 gene in myogenic cells significantly inhibited the cellular differentiation process [34]. Interestingly, LPAR1 was also enriched in the PI3K-AKT signalling pathway. These findings suggest that LPAR1 plays a crucial role in regulating the growth and development of muscles in Duroc pigs. SLIT3 in the H group exhibited hypomethylation in the downstream region and upregulated expression. Mice deficient in SLIT3 were reported to exhibit reduced skeletal muscle mass, muscle strength, and physical activity [35]. Luan M et al. discovered through WGAS that SLIT3 may have an effect on loin muscle area [36]. Upregulation of SLIT3 during the later growth stages of Duroc pigs may affect muscle development. The hypermethylation of MEF2C in the gene-body region and upregulated expression of this gene were found in the H group. MEF2C is a member of the myocyte enhancer factor 2 family and plays a role in myogenesis [37–39]. In recent years, an increasing number of comprehensive and systematic analyses of the role of MEF2C in myogenesis and muscle regeneration have been conducted. For example, Piasecka A. et al. reported that MEF2C is an essential factor regulating the quantity and quality of the microtranscriptome. Specifically, deleting MEF2C led to the downregulation of specific muscle-specific miRNAs during muscle cell differentiation [40]. Loumaye A et al. reported that MEF2C was able to maintain the slow expression and protein content of the myosin heavy chain beta(MyHC-$\beta$) subtype in differentiated myotubes [41]. Moreover, Kim HB et al. (2020) reported that O-GlcNAcylation of MEF2C was necessary for regulation of myoblast differentiation [42]. Interestingly, MEF2C was also enriched in the MAPK signalling pathway. Overall, MEF2C might also be a master regulator in Duroc pigs with different growth rates. Thus, our study provides data support and new research ideas for exploring genes related to skeletal muscle growth and development in Duroc pigs.

## Conclusion

In conclusion, Our study demonstrates that the methylation status affects the growth rate of pigs and the expression level of genes related to muscle growth and development.

## Materials and methods

**Animals**

All animal care and treatment procedures were approved by Ethics Committee of Shandong Agricultural University, China, and performed according to the Committee’s guidelines and regulations (Approval No.: 2004006). Duroc pigs came from a core breeding farm (our study has taken the informed consent of the animal owner), with the measurement data in the pig herd to 30 to 110 kg body weight (individuals in the top 30% of ADG), and the performance measurement was continued to about 130 kg body weight. According to the ADG (Supplementary Table 5), eight pigs were selected and divided into two groups: the H (774.89 g) group and the L (658.77 g) group, and each pair of high and low groups were half-siblings. All pigs were humanely slaughtered by electronic stunning followed by exsanguinations at the local abattoir. The LDM tissues were sampled and snap-frozen in liquid nitrogen, and stored at -80 ^∘^C for later use.

**DNA isolation, BS-seq library construction, and sequencing**

High-quality genomic DNA was extracted from the LDM using DNeasy Blood & Tissue Kits (QIAGEN, CA, USA). DNA concentration and integrity were c Agarose Gel Electrophoresis, respectively. Then, the DNA libraries for bisulfite sequencing were prepared. Briefly, genomic DNAs were fragmented into 100-300 bp by Sonication (Covaris, Massachusetts, USA) and purified with a MiniElute PCR Purification Kit (QIAGEN, MD, USA). The fragmented DNAs were end-repaired, and a single “A” nucleotide was added to the three ^′^ ends of the blunt fragments. Then, the genomic fragments were ligated to methylated sequencing adapters. Chips with adapters were bisulfite converted using a Methylation-Gold kit (ZYMO, CA, USA), and unmethylated cytosine was converted to uracil during sodium bisulfite treatment. Finally, the altered DNA fragments were PCR amplified and sequenced using Illumina HiSeq™ 2500 by Gene Denovo Biotechnology Co. Ltd. (Guangzhou, China).

**BS-seq reads mapping and methylation level analysis**

To get high-quality clean reads, raw reads were filtered according to the following rules: 1) removing reads containing more than 10% of unknown nucleotides (N); 2) removing low-quality reads containing more than 40% of low-quality (Q-value $\le 20$) bases.

By default, the obtained clean reads were mapped to the species reference genome Sus scrofa v. 11.1 using BSMAP software [43] (version: 2.90). Then a custom Perl script was used to call methylated cytosines and the methylated cytosines were tested with the correction algorithm described by [44]. The methylation level was calculated based on methylated cytosine percentage in the whole genome, in each chromosome, and in different regions for each sequence context (CG, CHG, and CHH). Additionally, the methylation profile at flanking 3-kb regions and the gene-body (or transposable elements) was plotted according to the average methylation levels of each 200-bp interval to evaluate different methylation patterns in other genomic regions.

**Differentially methylated regions (DMRs) and functional enrichment analysis**

To identify differentially methylated regions (DMRs) between two samples(Methyl Kit (V1.4.10)), the minimum read coverage to call a methylation status for a base was set to 3 DMRs for each sequence context (CG, CHG, and CHH) according to different criteria: 1) For CG, numbers of CG in each window $\ge 5$, the absolute value of the difference in methylation ratio $\ge 0.15$, and P $\le 0.05$; 2) For CHG, numbers in a window $\ge 5$, the absolute value of the difference in methylation ratio $\ge 0.25$, and Q $\le 0.05$; 3) For CHH, numbers in a window $\ge 15$, absolute value of the difference in methylation ratio $\ge 0.15$, and Q $\le 0.05$; 4) For all C, numbers in a window $\ge 20$, absolute value of the difference in methylation ratio $\ge 0.2$, and Q $\le 0.05$. To analyze the functional enrichment of genes affected by DMRs, gene ontology (GO) enrichment analysis (http://www.geneontology.org/) and KEGG pathway enrichment analysis (http://www.kegg.jp/kegg/) were conducted for DMGs by the hypergeometric test with a corrected *p*-value $\le 0.05$.

**Bisulfite sequencing PCR**

Methprimer-designed BSP primers, which are mentioned in Table 2. Using a BisulFlashTM DNA Modification Kit (Epigentek, Farmingdale, USA), the bisulfite conversion of isolated LDM genomic DNA was shown. The targeted portion was amplified by PCR using TaKaRa EpiTaq™ HS (TaKaRa, Japan). The PCR products were cloned into the pMD18-T vector (TaKaRa, Japan) and transformed into Escherichia coli DH5$\alpha$ competent cells (TaKaRa, Japan). Thirty positive clones were sequenced for each group. Site-specific methylation measurements were analyzed using QUMA-Analyzer.

**RNA extraction and qRT-PCR**

High quality RNA was extracted from the LDMs using a RNA extraction kit (Tiangen, China). First-strand cDNA was synthesized using the PrimeScript RT reagent Kit (TaKaRa, Japan). The primers for qPCR were designed using Primer Premier 6.0 and were listed in Table 2. The qRT-PCR assays were performed in a $20 \mu$L reaction volume on a Roche LightCycler® 96 with TB Green as the fluorescent dye according to the manufacturer’s instructions (TaKaRa, Japan). After normalization with GAPDH,relative RNA levels in samples were calculated by the comparative threshold cycle (Ct) method.

**Integrative analysis of transcriptome and WGBS data**

To investigate the relationship between DNA methylation and gene expression (the same batch of Duroc pigs was used for RNA-Seq and WGBS), all DMGs were divided into three groups based on DMR location (upstream, gene body, and downstream), and a Venn diagram was used to visually demonstrate the overlap of genes between DMGs and DEGs.

**Statistical analysis**

The qPCR data were analyzed by the one-way ANOVA model followed by Tukey’s multiple range tests to separate the means using the SAS computer program for Windows (version 9.2). Data were presented as means ± SDs, and the statistical significance was set at *P* < 0.05.

### Supplementary Information

Supplementary Material 1.

## Data Availability

The RNA-seq raw data are available at: https://www.ncbi.nlm.nih.gov/bioproject/?term=PRJNA812354. The WGBS raw data are available at: https://www.ncbi.nlm.nih.gov/bioproject/?term=PRJNA1054366.
